# Development of a standard set of PROs and generic PROMs for Dutch medical specialist care: Recommendations from the Outcome-Based Healthcare Program Working Group Generic PROMs

**DOI:** 10.1007/s11136-022-03328-3

**Published:** 2023-02-09

**Authors:** Martijn Oude Voshaar, Caroline B. Terwee, Lotte Haverman, Bas van der Kolk, Marleen Harkes, Christiaan S. van Woerden, Fenna van Breda, Stephanie Breukink, Irma de Hoop, Hester Vermeulen, Evelien de Graaf, Jan Hazelzet, Barbara van Leiden, Jozette Stienen, Marian Hoekstra, Hans Bart, Hester van Bommel, Domino Determann, Mariët Verburg, Philip van der Wees, Anna J. Beurskens

**Affiliations:** 1 National Health Care Institute (Zorginstituut Nederland), Diemen, The Netherlands; 2 Department of Medical Cell BioPhysics & TechMed Center, University of Twente, Enschede, The Netherlands; 3 Epidemiology and Data Science, Amsterdam UMC Location Vrije Universiteit, Amsterdam, the Netherlands; 4 Methodology, Amsterdam Public Health Research Institute, Amsterdam, The Netherlands; 5 Dutch Society of Medical Specialists, Amphia Hospital, Utrecht, The Netherlands; 6 Dutch Nurses’ Association, Amphia Hospital, Utrecht, The Netherlands; 7 Dutch Federation of University Medical Centres, Utrecht, The Netherlands; 8 National Association of Dutch Health Insurers, Zeist, The Netherlands; 9 Dutch Hospital Association &, Amphia Hospital, Breda, The Netherlands; 10 Private Clinics Netherlands, Zoetermeer, The Netherlands; 11 The Netherlands Patients Federation, Utrecht, The Netherlands; 12 Pharos - Dutch Centre of Expertise On Health Disparities, Utrecht, The Netherlands; 13 Department of Family Medicine, CAPHRI School for Public Health and Primary Care, Maastricht University, Maastricht, The Netherlands; 14 Radboud University Medical Center, Radboud Institute for Health Sciences, IQ Healthcare and Department of Rehabilitation, Nijmegen, The Netherlands

**Keywords:** Patient reported outcomes (PROs), Patient reported outcome measures (PROMs), Value-based health care (VBHC), Shared decision-making (SDM), Quality improvement

## Abstract

**Purpose:**

The added value of measuring patient-reported outcomes (PROs) for delivering patient-centered care and assessment of healthcare quality is increasingly evident. However, healthcare system wide data collection initiatives are hampered by the proliferation of patient-reported outcome measures (PROMs) and conflicting data collection standards. As part of a national initiative of the Dutch Ministry of Health, Welfare and Sport we developed a consensus-based standard set of generic PROs and PROMs to be implemented across Dutch medical specialist care.

**Methods:**

A working group of mandated representatives of umbrella organizations involved in Dutch medical specialist care, together with PROM experts and patient organizations worked through a structured, consensus-driven co-creation process. This included literature reviews, online expert and working group meetings, and feedback from national patient- and umbrella organizations. The ‘PROM-cycle’ methodology was used to select feasible, valid, and reliable PROMs to obtain domain scores for each of the PROs included in the set.

**Results:**

Eight PROs across different domains of health were ultimately endorsed: symptoms (pain & fatigue), functioning (physical, social/participation, mental [anxiety & depression]), and overarching (quality of life & perceived overall health). A limited number of generic PROMs was endorsed. PROMIS short forms were selected as the preferred instruments for all PROs. Several recommendations were formulated to facilitate healthcare system level adoption and implementation of the standard set.

**Conclusions:**

We developed a consensus-based standard set of Generic PROMs and a set of recommendations to facilitate healthcare system wide implementation across Dutch medical specialist care.

**Supplementary Information:**

The online version of this article (10.1007/s11136-022-03328-3) contains supplementary material, which is available to authorized users.

## Plain English summary

1. What is the key problem/issue/question this manuscript addresses?

Routine assessment of patients' own perceptions of their health related quality of life has become increasingly integrated in daily clinical practice. The value of the resulting data for performance assessment, helping patients make informed decisions about their care and other purposes is increasingly realized by different stakeholders.

2. Why is this study needed?

The widespread adoption of Patient reported outcome measures (PROMs) in various data collection initiatives within the healthcare system has resulted in many valuable insights. However, outcomes are frequently difficult to compare between such initiatives because different PROMs are included.

3. What is the main point of your study?

This study describes the working process and recommendation of a working group initiated by the Outcome-Based Healthcare Program that set out to align PROM data collection in Dutch medical specialist care by developing a Standard Set of generic Patient reported outcomes and PROMs.

4. Provide a brief overview of your results and what they mean.

The working group recommended routine collection of outcomes in 8 health domains. PROMIS instruments are the preferred instruments to assess these outcomes. However, other PROMs that meet a number of psychometric standards and that are linked to the respective PROMIS metrics can also be used.

## Introduction

An increasing number of national health authorities recommend routine collection of Patient Reported Outcomes (PROs) from patients as they undergo specialist care. It has previously been shown that patient level PRO data can be used in daily care settings to better involve patients in medical decisions [1–3], while data aggregated across providers can be used for quality improvement by benchmarking, shared learning and shared decision-making support tools [4, 5]. Aggregated PROM data may also be used to provide real-world evidence about treatment effectiveness and safety [6, 7], and accountability to payers and the public [8].

In many countries, PROMs are included in data collection initiatives for one or more of these purposes. However, such initiatives typically operate independently and focus on single or a limited number of care processes. While the resulting data allow outcomes to be compared among providers that offer similar services, unaligned data standards and the proliferation of different PROMs often precludes data to be shared between healthcare services [9–11]. Consequently, outcomes can usually not be assessed across the entire chain of care and multi-morbid patients receiving care from multiple medical specialists might have to complete different PROMs for the different healthcare services [12, 13].

One reason for the proliferation of PROMs within the healthcare system is that condition centric international recommendations are often followed for PRO and PROM selection. These are developed by independent working groups and for different patient populations. For example, an analysis of the International Consortium of Health Outcomes Measurement (ICHOM) Standard Sets published up to June 2021 shows that 114 PROMs have thus far been recommended for use in the 39 conditions for which a Standard Set was developed. However, content analyses of these PROMs show that they typically focus on a limited number of generic concepts, in particular the ability to carry out daily activities, participate in social roles, and the presence and severity of common symptoms such as pain and fatigue [14, 15]. This suggest that problems associated with the proliferation of PROMs could be addressed by collecting a limited set of generic PROs that reflect common areas of disease impact from all patients in medical specialist care at regular intervals of time and supplement these with disease specific measures as needed. Once implemented, the resulting data could be shared among all stakeholders.

In the Netherlands, the umbrella organizations involved in medical specialist and the Ministry of Health, Welfare and Sports have jointly started the Outcome-Based Healthcare Program to better incorporate the measurement of health outcomes in daily medical specialist care, which was one of the policy goals agreed upon in the policy agreement medical specialist care 2019–2022. As part of this program, a working group of mandated representatives of the umbrella organizations and methodological, as well as PROM, experts were tasked with developing a standard set of generic PROs and PROMs. This set is intended to be implemented in daily management of patients and for quality assessment across a broad range of conditions. The objective set out in the working plan of the overall program was to develop a standard set that 1) consists of PROs and PROMs relevant across medical conditions, 2) is supported by all stakeholders, 3) is consistent with relevant ongoing PROM initiatives in The Netherlands and internationally, and 4) be minimally burdensome to patients and health professionals. This paper describes the working process, results, and recommendations of this working group.

## Methods

### Context and setting

The goals of the Dutch Outcome-Based Healthcare Program are to stimulate routine collection of patient level outcome data from patients undergoing specialist care to be used for shared decision-making, quality assessment, and promoting outcome-based organization and payment. To achieve these goals, sets of core outcomes are being developed for multiple medical conditions that together make up approximately 50% of the Dutch burden of disease according to the National Healthcare Institute [17]. Each set is developed by a condition specific working group and contains both clinical and patient reported outcomes. A pilot of the program was conducted in inflammatory bowel disease (IBD), chronic kidney disease (CKD), knee osteoarthritis (KOA) and pancreatic cancer. As described below, literature reviews that were conducted as part of this pilot phase were also used for selecting PROs to be included in the standard set.

### Composition of generic PROM working group

The working group consisted of 12 representatives of the umbrella organizations that signed the policy agreement Medical Specialist Care 2019–2022. These included organizations representing patients, medical specialists, nurses, health insurers, general hospitals and specialist institutions, university medical centers and independent clinics. To provide methodological and scientific input, the working group was complemented with a project team consisting of four methodologists and Dutch experts in the field of PROMs (CT, PvdW, MOV, LH) and one expert from the national center of expertise on health disparities. The working group was supported by a chair, secretary and three methodologists who conducted systematic literature reviews to guide and substantiate the decisions by the working group.

### Working group process

**Fig. 1:**
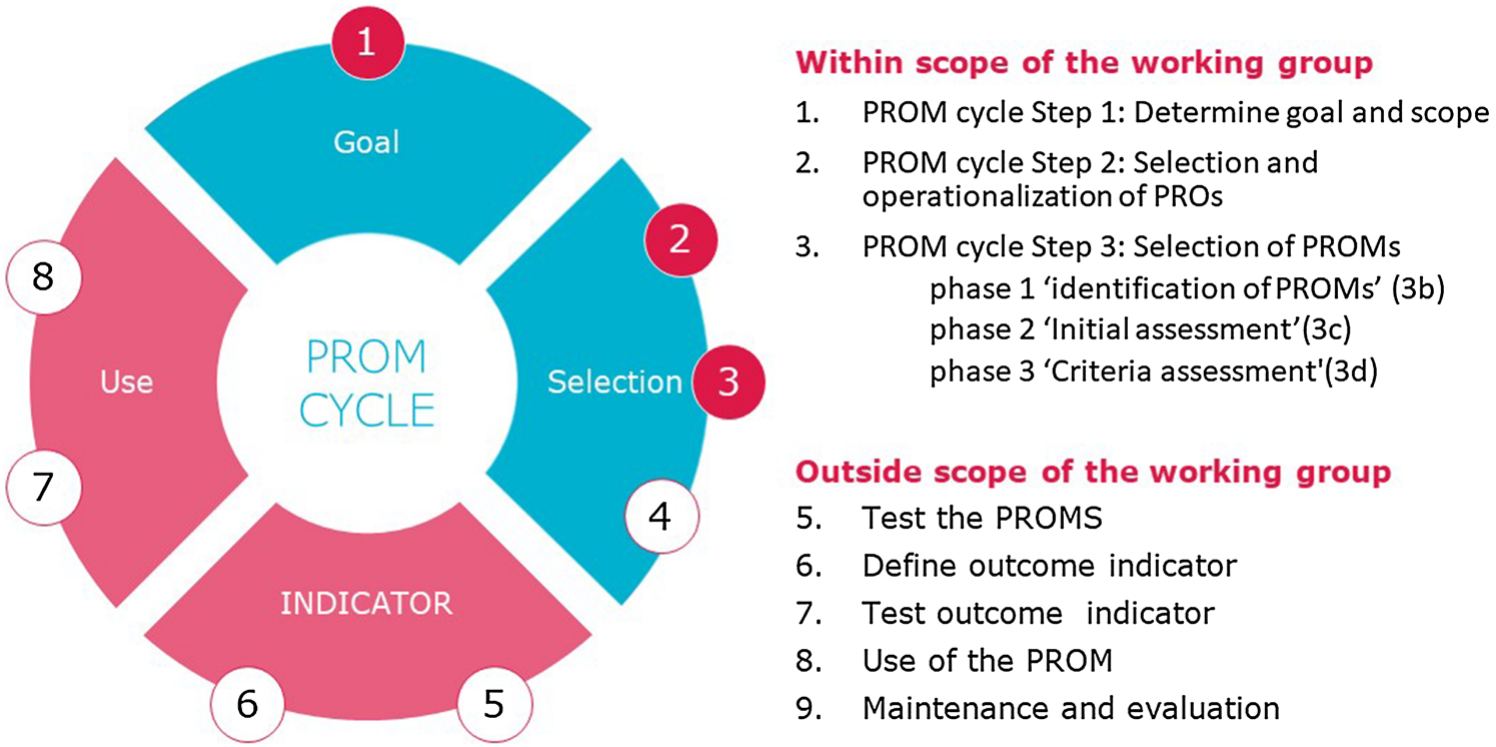
The PROM-cylce methodology in relation to the Working Group process

The standard set of generic PROs and PROMs was developed using a consensus driven, structured process in which the group worked in co-creation through the first three steps of the PROM-cycle, which is a framework to support the selection and implementation of PROMs [18]. Figure 1 maps the various activities performed by the working group into the steps of the PROM-cycle framework. The way in which these steps would be operationalized was pre-discussed and coordinated with the working group in all cases. These activities are described in more detail in the sections below. Over a period of approximately one year, seven online working sessions were held. The activities of each working session are summarized in Supplemental Fig. 1. Preceding most sessions literature searches were conducted to identify relevant information on PROs for working sessions related to PROM cycle step 2 and PROMs for working group sessions related to PROM cycle step 3, as described in more detail below. Also, the working group was provided with feedback collected from the umbrella organizations and patient organizations on 1) the proposed set of PROs and 2) the proposed set of PROMs to be included in the Standard Set. The project team and methodological experts used the collected information to prepare proposed content for the set. During the sessions, these proposals along with supporting information were presented to the working group for endorsement.

### PROM cycle step 1: determine goal and scope

Before the actual PRO and PROM selection, agreement of key concepts of PROs and selection criteria of PROMs was established. During the first two working sessions this was the scope and deliberately done before the final selection, to minimize the impact of subjective factors on the selection of PROs and PROMs to be included in the set.

### PROM cycle step 2: selection and operationalization of PROs

PROs to be considered for inclusion in the standard set were identified through various avenues. This included reviews of the PRO domain frameworks of the positive health measurement approach [19] PROMIS and previous recommendations by the Dutch Linnean initiative [20], PROs included in the 39 ICHOM sets according to the reference guides published on their website (consulted in April 2021), and the results of three independent, unpublished literature reviews of qualitative studies that had previously been performed to support the work of three condition specific OHP pilot working groups (IBD, CKD and KOA). In these reviews qualitative studies in which patients were asked to describe how their disease affected their daily lives, using an open-ended question format were reviewed. The reviews were performed according to methodology described elsewhere [16].

To help ensure that the ultimate set of PROs would allow for comprehensive assessment of health and to facilitate consensus, a presentation on conceptual frameworks for classifying PROs proposed by Wilson and Cleary, and expanded by Valdereras and Alonso was prepared by the methodological experts [15, 21]. In the works of these authors health outcomes are classified in a number of hierarchically related categories ranging from symptom status, functional status to general health perceptions and health related quality of life. PROs that had thus far been identified were mapped onto this framework by the working group and refined during one of the working sessions which led to a reduced set of 12 PROs (supplemental Table 2). To further condense this reduced list, working group members and 8 national patient advocacy groups were each asked to select the 7 most important PROs from this list. The patient advocacy groups were recruited via e-mail by The Netherlands Patients Federation. oO guide final PRO selection, the PROs were ranked by the number of times each PRO was included in the top 7 by the patient advocacy groups and by the working group members.

The PRO framework was then operationalized by the methodological experts in terms of subdomains, if any, that make up each PRO domain and preferred ways of measuring them with respect to item format and time frame. Feedback on the final set of PROs was obtained from each of the umbrella organizations both in writing and via videoconference. Additional feedback on the relevance of each PRO to patients was obtained during a webinar among > 20 Dutch patient organizations organized by the Netherlands Patient Federation.

### PROM cycle step 3: selection of PROMs

In phase 1 ‘identification of PROMs’, a comprehensive list of eligible PROMs or relevant subscales of PROMs was compiled, i.e. a longlist of PROMs or PROM subscales. These were identified in the various initiatives that were reviewed for PRO selection in the previous step. In addition, we consulted a systematic review on PROMs included in core sets in the COMET database [22]. Finally, we included all PROMs that were used to validate the widely used generic PROMIS item banks against in the PROMIS Wave 1 testing initiative [23]. The resulting list was expanded via a survey among the umbrella organizations and working group members.

In phase 2 ‘Initial assessment’, three expert PROM researchers (CT, PvdW, MOV) assessed all PROMs on the list for face validity by answering two questions: 1) Is the PROM a generic measure? and 2) Does the PROM measure any of the endorsed PROs in a way that is consistent with the operationalization proposed by the working group? If all three experts agreed the answer to either question was no, the PROM was excluded from further review.

In phase 3 ‘Criteria assessment’, the remaining (subscales of) PROMs were subjected to a detailed review of quality criteria that were established by the working group in PROM cycle step 1: content validity, feasibility to implement, measurement properties, and the possibility to express scores in or convert scores to an item response theory (IRT) based standardized score metric. Two aspects of content validity (relevance and comprehensiveness) were rated by two experts (CT, MOV) using the COSMIN criteria for content validity [24]. The criteria understandability and accessibility for patients were assessed by the ‘Pharos rapid test for patient questionnaires’ which is a checklist developed to evaluate the suitability of questionnaires for people with low health literacy, developed by Pharos, the Dutch center of expertise on health disparities. Feasibility was further evaluated by considering license costs, and number of items. A systematic review of the evidence on measurement properties of the remaining PROMs was conducted. We used a sensitive search filter for finding studies on measurement properties of the relevant PROMs [25] and applied slightly adapted versions of the criteria for good measurement properties listed in the COSMIN manual for systematic reviews of PROMs and ISOQOL criteria to rate the measurement properties of included PROMs [26]. In general, this review was limited to the four pilot conditions of the Outcomes-Based Healthcare Program, since the measurement properties of well-known generic PROMs were expected to have been evaluated in an unmanageable number of papers. However, for several PROMs in widespread use in The Netherlands (TOPICS-SF, Distress thermometer and Positive Health Measurement Tool) all studies on measurement properties were reviewed. To assess the evidence for construct validity, all correlations with other PROMs were extracted and tested against pre-specified hypotheses about the expected correlations between common PROs (results available on request via first author).

For each of the selection criteria determined by the working group in PROM cycle step 1, ‘signaling questions’ were formulated, inspired by the OMERACT Filter 2.1 for instrument selection, in such a way that each PROM could receive a ‘green’, ‘amber’, or ‘red’ rating for each criterion [27]. The criteria were structured to ensure that all PROMs which received green ratings for all criteria would be endorsed for inclusion. PROMs which received one or more amber ratings, but no red ratings would be provisionally endorsed and PROMs with one or more red ratings would not be endorsed. A detailed overview of the signaling questions is provided in Supplemental Table 1.

In phase 4 “stakeholder feedback’ a report with the preliminary results was submitted for feedback to the members of each umbrella organizations, including 10 disease specific patient advocacy groups. We obtained written feedback from 34 member originations. Feedback on the proposed set of PROMs was also obtained during an invitational webinar among 95 professionals recruited via the umbrella organizations. Based on the obtained feedback the working group made final recommendations on the content and application of the standard set.

## Results

### PROM cycle step 1: determine goal and scope

It was decided that the development of the set should be guided by several principles. Firstly, the set should be restricted to a minimum number of PROs that are relevant across medical specialist care conditions. It was decided that overarching PRO domains (such as overall health or quality of life), if included, should be assessed using single item PROMs, to avoid overlap with more specific domains (such as physical function). Furthermore, it was decided it should be possible to obtain valid and reliable domain scores for each individual PRO included in the set. Therefore, PROMs in which items reflecting different domains are combined in a single score (such as the PROMIS Scale v1.2- Global Health) were excluded from consideration. Finally, it was decided that for each PRO, multiple PROMs could be recommended, if scores could be converted to a common metric using IRT methodology. Based on the results of a systematic literature search and expert opinion it was decided that the PROMIS T-score metrics would be the most useful metrics for this because many generic PROMs have already been linked to these score metrics. Therefore, this was added as a PROM selection criterion.

### PROM cycle step 2: selection and operationalization of PROs

60 PROs were identified that the working group initially organized into the broad domains of physical, mental, and social functioning, symptoms, and overarching health outcomes (Supplemental Table 2). Within these broad domains 12 conceptually distinct PROs were identified that might be sufficiently relevant to include in the standard set. The working group members as well as eight national patient organizations were then asked to select the most important domains from this list. The experts used the resulting data to propose a standard set of eight PROs which was subsequently endorsed by the working group. The definitions and subdomains of each PRO included in the set are presented in Table 1.

**Table 1:** Final selection of PROs

	PRO domain		Specification	Subdomains
Symptoms	Fatigue		Degree (intensity) of fatigue	None
	Pain		Single item rating of degree (intensity) of pain	None
Functioning	Physical functioning		Ability to perform everyday activities	1. Activities of daily living 2. Instrumental activities of daily living 3. Mobility
	Participation in social roles		Ability to participate in social roles and activities	1. Engaging in formal and informal relationships 2. Engaging in family roles 3. Engaging in domestic roles 4. Engaging in work or education related roles 5. General
	Mental functioning	Anxiety	Experienced anxiety	1. Cognitive anxiety: Anticipating acute (fear) or future (anxiety) threats 2. Physiological anxiety: Experiencing anxiety related bodily sensations including increased heart rate and restlessness
		Depression	Experienced depressive symptoms	1. Dysphoria (sadness, irritability) 2. Anhedonia (loss of interest or pleasure in activities of daily living) 3. Disruption of vegetative functions (psychomotor retardation, insomnia, loss of appetite)
Overarching	Perceived health		Single item rating of perceived overall health	None
	Quality of life		Single item rating of perceived overall quality of life	None

### PROM cycle step 3: selection of PROMs

**Fig. 2:**
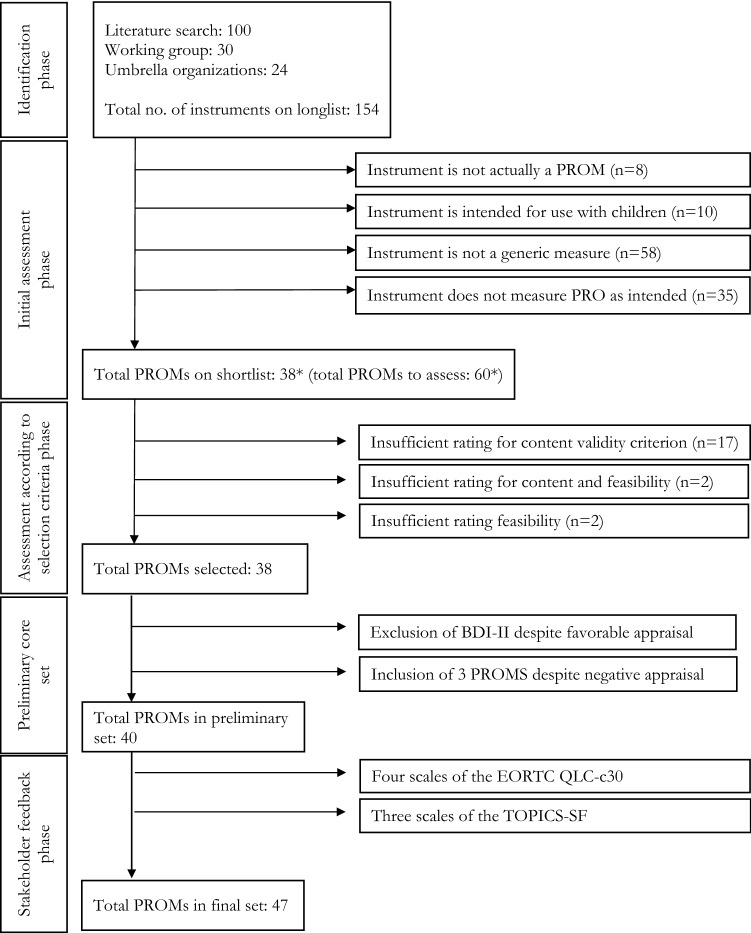
PROM selection flow chart. *PROMIS instruments derived from the same item bank were subsumed under a single entry on the short list. Each individual PROMIS short-form and CAT was evaluated separately in the critical evaluation phase

During the ‘identification’ phase, we identified a total of 154 PROMs. In phase 2 ‘initial assessment phase’, 114 of the PROMs were excluded and not considered for further review (Fig. 2). Main reasons were: PROMs were not generic (58), lacked face validity (35), PROMs were specifically developed for children (10), or were judged to not be PROMs (8) either because they were intended to be scored by healthcare professionals or because responses of patients were weighted or otherwise manipulated (e.g. preference based measures to obtain health utilities).

Supplemental Table 3 presents an overview of the results of phase 3 ‘critical assessment’. Insufficient content validity was the most common reason to exclude PROMs. Four PROMs received an insufficient rating for feasibility all due to insufficiently understandable and/or accessible for patients according to the Pharos rapid test. Furthermore, it was noted that only eight PROMs received a favorable rating for understandability/accessibility domains of feasibility. Supplemental Table 3 on available evidence regarding measurement properties of the shortlisted PROMs in the pilot condition summarizes the findings of 154 papers identified in the systematic Pubmed search. The shortlisted PROMs were generally found to have sufficient measurement properties. All indeterminate ratings resulted from an insufficient amount of evidence that was nevertheless reflective of good measurement properties.

The results were presented to the working group for endorsement (phase 4). The working group agreed with the results of the PROM selection process overall. However, the Beck Depression Inventory was judged to not be suitable for the intended applications of the standard set as its content generally reflects severe symptoms of depression. Furthermore, the PROMIS Physical Function short form 8b was included despite a negative rating for content validity (because no items on self-care are included) because the working group considered it important that PROMs with less than 10 items would also be available to assess physical function. Finally, the SF-36 Bodily Pain and Sf-36 Emotional Role functioning scales were ultimately not excluded despite negative ratings. The SF-36 former received a negative rating for feasibility due to one item having too many response options and the latter because of slightly complicated syntax.

In the final ‘stakeholder feedback’ phase of the PROM selection process, two major recommendations were made. Multiple stakeholders expected more guidance from the working group in the form of the recommendation of a single PROM to assess each of the endorsed PROs. Other stakeholders, argued for the inclusion of the TOPICS-SF and the EORTC QLQ-c30 which are both widely used in the Netherlands within elderly and oncological patients, respectively. The EORTC QLQ- C30 was excluded in the ‘ initial assessment phase (phase 2 of PROM cycle step 3: selection of PROMs) because the methodological experts had judged this PROM to be unsuitable for use in non-oncological settings and therefore judged to be insufficiently generic to be put on the ‘PROM short-list’. The majority of the TOPICS-SF scales had been excluded because they did not receive favorable ratings for content validity in PROM cycle step 3 phase 3 ‘Criteria assessment’. Many stakeholders also expressed concern with respect to implementation and the technical feasibility of integrating PROM data in the daily workflow. However, this was outside the scope of the Generic PROM working group?

### Recommendations of the Generic PROM working group

The final set of endorsed PROMs is presented in Table 2. In response to stakeholder feedback the working group decided to include two additional scales of the TOPICS-SF that had initially received a doubtful rating for content validity and four scales of the EORTC QLQ-c30 in the standard set, even though these PROMs were initially excluded. Furthermore, to assist new adopters of PROMs the working group selected a preferred PROM for each PRO (highlighted PROMs in Table 2). This set of preferred PROMs was selected to maximize feasibility. PROMIS short forms were selected as the preferred instruments for all PROs [28]. These short forms are available free of charge and contain the minimum possible number of items, while retaining most minimum standards in terms of measurement properties. However, in situations where precise PROM scores are especially important, for example when treatment decisions are based on individual PROM scores, longer PROMIS short-forms or PROMIS computerized adaptive tests (CATs) are recommended.

**Table 2:** Final selection of PROMs

	**PRO(s)**		**Generic PROM(s)**	**# items**
Overarching	Quality of life		**PROMIS® Global02**	1
			TOPICS-SF NRS Quality of life*	1
			EORTC QLQ-c30-v3 Quality of life (item 30)**	1
	Perceived health		**PROMIS® Global01** / SF-36—question 1	1
			TOPICS-SF NRS Overall health*	1
			EORTC QLQ-c30 v3 Perceived health (item 29)**	1
Functioning	Participation in social roles		**PROMIS® Ability to Participate in Social Roles and Activities** [**4a** / 6a / 8a / CAT]***	4–12
			SF-36/RAND-36 Role Functioning (emotional and physical problems)	7 (3 + 4)
			Utrecht Scale for Evaluation of Rehabilitation-Participation (USER-Participation)v	11
	Physical functioning		**PROMIS® Physical Function** [**8b** / 10a / 10b / 20a / CAT]	4–20
			SF-36/RAND-36 Physical Functioning	7 (3 + 4)
			TOPICS-SF Tasks and activities of daily living*	10
			EORTC-QLCc30 v3 Physical Functioning**	5
	Mental functioning	Anxiety	**PROMIS® Anxiety 4a** / 6a / 8a / 7a/ CAT]***	4–12
			Generalized Anxiety Disorder -7 (GAD-7)	7
			Four Dimensional Symptom Questionnaire (4DKL)—Anxiety	12
			Hospital Anxiety and Depression Scale (HADS)—Anxiety	7
		Depression	**PROMIS® Depression 4a** / 6a / 8a / 8b / CAT***	4–12
			Center for Epidemiologic Studies Depression (CES-D)	20
			Patient Health Questionnaire (PHQ) [2 / 9]	2 or 9
			Four Dimensional Symptom Questionnaire (4DKL)—Depression	6
Symptoms	Fatigue		**PROMIS® Fatigue 4a** / 6a / 8a / CAT***	4–12
			EORTC-QLQ-c30 v3 Fatigue**	3
			Numeric Rating Scale Fatigue (NRS-Fatigue)	1
	Pain		**Numeric Rating Scale Pain Intensity (NRS-Pain intensity)**	1
			SF-36/RAND-36 Bodily Pain	2

Preferred PROMs for each PRO are highlighted in bold

PROMs highlighted in bold constitute the set of PROMs preferred by the working group

*For frail elderly

**For cancer patients without co-morbidities

***When using PROMIS-measuring instruments one can choose from a number of short-forms or CATS with a varying number of questions

****Both scales of SF-36/RAND-36 are needed to measure the PRO-construct Participation in social roles

Based on the results of the PRO and PROM selection processes, and feedback obtained from the umbrella organizations, the working group also formulated several recommendations for how the standard set of generic PROMs should be used by specialist care providers and further developed in The Netherlands. Firstly, the working group recommends that data collected using the Standard Set is discussed during clinic visits with patients and, in so far appropriate, considered when making medical decisions. Aggregated PROM data should be used for healthcare quality assessment, shared learning and shared decision-making support tools. Secondly, the time intervals at which PROM data are collected should be limited to clinically important time periods. Thirdly, for certain conditions it may be desirable to supplement the standard set with disease-specific PROMs and/or PROMs that measure other PROs. In these cases, the overall burden for patients should be carefully considered and overlap in PROM content should be avoided. Fourthly, further work is necessary to ensure that PROM data can be shared between stakeholders and outcomes can be assessed across the full chain of care. Fifthly, a national governance framework should be developed to facilitate healthcare system level technical implementation and continuity of the set. Sixthly, experiences with the set should periodically be reviewed and the set updated as required. Finally, further practical applications of the set of generic PROMs should be actively pursued.

The Standard set of Generic PROs and PROMs and these recommendations were formally endorsed by the boards of all involved umbrella organizations in June 2022.

## Discussion

In this project, a working group of mandated representatives of the umbrella organizations involved in Dutch medical specialist care collaborated with experts and disease specific patient advocacy groups to develop a consensus and evidence-based standard set of generic PROMs. This set is to be implemented in the daily medical specialist care for Dutch patients.

The standard set was carefully designed to include a limited set of PROs relevant across most medical conditions in medical specialist care and different levels of health impact. The set of PROs allows for a comprehensive assessment of generic symptoms, physical, mental, and social function, and the overarching concepts of perceived health and overall quality of life. Multiple well-known generic PROMs or subscales of PROMs with high content validity, sufficient measurement properties and that should be usable by all patients, including those with low literacy have been endorsed to assess each of the PROs However, none of the currently included PROMs met all the standards we set for acceptability and understandability. Since patients with lower health literacy might face difficulties completing the currently endorsed PROMs, particular attention should be paid to acceptability and understandability of newly developed generic PROMS for future revisions of the Standard Set.

The working group recommended that the endorsed PROs are collected from all patients in medical specialist care and that this set may be expanded by disease specific PROMs as needed. This approach was also chosen for the Welsh PROMs, PREMs and Effectiveness Program (PPEP) [29]. PPEP aims to collect the EQ-5D-5L and WPAI from all patients in secondary care patients across Wales and supplements these with condition specific PROMs. The inclusion of two generic instruments allows outcomes to be compared among all those who implement PPEP. Achieving comparability by limiting the number of PROMs to one per PRO was considered infeasible for the Standard Set described in this work; Many different PROMs are either in use already or supported by individual initiatives in medical specialist care in The Netherlands and one of the requirements set by umbrella organizations was that the Standard Set should receive approval from all stakeholders. The decision to include multiple PROMs for a number of PROs, provided these PROMS were derived from or linked to the respective PROMIS item banks, was motivated by the desirability of being able to include the maximum number of well-known PROMs while retaining standardization of measurement. An unintended consequence of this was that it limited the potential for PROMs other than PROMIS to be included in the Standard Set and contributed to the PROMIS instruments being recommended as favored instruments. To address this, several PROMs were included in the standard set have not yet been linked to the relevant PROMIS T-score metric. In all cases, these PROMs met none of the exclusion criteria and were considered important to include either by the working group or other stakeholders in Dutch Medical Specialist Care. Several Project are now ongoing in which crosswalks are being developed to address this limitation.

While we recommend that users of the standard set make use of the PROMIS T-score metric for all intended uses involving aggregated data, IRT based linking procedures are intended for group level score conversions [30]. Requirements for interchangeability of scores on individual level are much more stringent. Future studies are required to examine, which, if any, PROMs can be ‘equated’ and thus used interchangeably also at the individual level [31].

In contrast to many earlier initiatives, we deliberately chose an unstructured consensus-based co-creation approach instead of the commonly used Delphi approach. A variety of methods were used in which working members and experts could share and discuss perspectives in a safe and open way. The approach was also theory driven through the PRO and PROM selection process in accordance with the PROM cycle methodology and COSMIN selection criteria, while working group members were asked to consider the practical implications and needs and values of the umbrella organizations they represented at each step of the process. As part of this way of working, a set of criteria for PROM selection was co-created by the methodological experts and working group members that is both methodologically rigorous and sensitive to the many well-described barriers to PROM data collection in daily practice [32, 33] We believe that investing in sharing views and knowledge and using predefined selection criteria for PROMs increased the generalizability and acceptability of our findings compared with more structured consensus building approaches such as the Delphi method. Although other working groups might have proposed different criteria, the criteria that were developed as part of this project align well with widely accepted international standards for PROM evaluation and selection [34]. Finally, while the Standard Set was compiled for use in second line care, the included PROs should be relevant for implementation in primary care and be compatible.

This work also has some limitations. We were unable to review the measurement properties of all included PROMs across all conditions. However, although we maintain that it is important to evaluate the measurement properties of PROMs when they are first applied in new populations, measurement properties of PROMs depend on the condition they are applied in only to a certain extent. Factors such as the number of items and response options, the intrinsic relations between constructs being measured by the PROM, and the clarity of the questions are also important [35]. The results of our literature review therefore provide important yet incomplete validity and reliability evidence of the included PROMs. We recommend that adopters of this set examine the available evidence supporting the measurement properties of individual PROMs in the condition in which they wish to implement the standard set.

In conclusion, we present a carefully selected set of generic PROs and PROMs for use in daily medical specialist care and relevant across different patient populations and across different levels of health impact. Furthermore, we provided several recommendations to help ensure successful healthcare system level implementation of the set.

## Supplementary Information

Below is the link to the electronic supplementary material.

(PDF 794 kb)

## Data Availability

Not applicable.
