# Correction: Development of a standard set of PROs and generic PROMs for Dutch medical specialist care

**DOI:** 10.1007/s11136-023-03412-2

**Published:** 2023-04-06

**Authors:** Martijn Oude Voshaar, Caroline B. Terwee, Lotte Haverman, Bas van der Kolk, Marleen Harkes, Christiaan S. van Woerden, Fenna van Breda, Stephanie Breukink, Irma de Hoop, Hester Vermeulen, Evelien de Graaf, Jan Hazelzet, Barbara van Leiden, Jozette Stienen, Marian Hoekstra, Hans Bart, Hester van Bommel, Domino Determann, Mariët Verburg, Philip van der Wees, Anna J. Beurskens

**Affiliations:** 1 National Health Care Institute (Zorginstituut Nederland), Diemen, The Netherlands; 2 Department of Medical Cell BioPhysics & TechMed Center, University of Twente, Enschede, The Netherlands; 3 Epidemiology and Data Science, Amsterdam UMC Location Vrije Universiteit, Amsterdam, the Netherlands; 4 Methodology, Amsterdam Public Health Research Institute, Amsterdam, The Netherlands; 5 Dutch Society of Medical Specialists, Amphia Hospital, Utrecht, The Netherlands; 6 Dutch Nurses’ Association, Amphia Hospital, Utrecht, The Netherlands; 7 Dutch Federation of University Medical Centres, Utrecht, The Netherlands; 8 National Association of Dutch Health Insurers, Zeist, The Netherlands; 9 Dutch Hospital Association &, Amphia Hospital, Breda, The Netherlands; 10 Private Clinics Netherlands, Zoetermeer, The Netherlands; 11 The Netherlands Patients Federation, Utrecht, The Netherlands; 12 Pharos - Dutch Centre of Expertise On Health Disparities, Utrecht, The Netherlands; 13 Department of Family Medicine, CAPHRI School for Public Health and Primary Care, Maastricht University, Maastricht, The Netherlands; 14 Radboud University Medical Center, Radboud Institute for Health Sciences, IQ Healthcare and Department of Rehabilitation, Nijmegen, The Netherlands

**Correction to: Quality of Life Research** https://doi.org/10.1007/s11136-022-03328-3

In the original publication, one of the co-author names was mistakenly misspelled as "Jan Hazelet" instead of "Jan Hazelzet". It is updated in this correction.

