# Supplementary material for: Development of a standard set of PROs and generic PROMs for Dutch medical specialist care: Recommendations from the Outcome-Based Healthcare Program Working Group Generic PROMs
Source: Qual Life Res. 2023 Feb 9;32(6):1595–605. doi: 10.1007/s11136-022-03328-3 (PMC10172289; doi:10.1007/s11136-022-03328-3)
Supplement: Supplementary file 1 — (PDF 794 kb) [file 11136_2022_3328_MOESM1_ESM.pdf]

*This document contains supplementary material to the following paper: Development of a standard set of PROs and generic PROMs for Dutch medical specialist care. A consensus-base co-creation approach by Oude Voshaar et al.*

In Supplemental Figure 1, the activities of each working session are summarized.

Supplemental Table 1 contains signaling questions that were used in phase 3 of the study “criteria assessment” during which selected PROMs were subjected to a detailed review of quality critieria. These questions were inspired by the OMERACT Filter 2.1 for instrument selection, in such a way that each PROM could receive a ‘green’, ‘amber’, or ‘red’ rating for each criterion. Supplemental Table 3 presents an overview of the results of this phase 3.

Supplemental Table 2 summarizes the results of the PRO selection step of PROM cycle step 2 PRO selection and operationalization

**Supplemental Figure 1. Activities per working session**

| | | | | | | | | |
|---|---|---|---|---|---|---|---|---|
| | 1 | 2 | 3 | 4 | 5 | 6 | | 7 |
| | Dec '20 | Febr '21 | March '21 | Apr '21 | May '21 | Jun '21 | | Dec '21 |
| Step | Kick-off | Objective and criteria | Selection PROs | Selection PRO(M)s | Selection PROMs | Selection PROMs | | |
| | Session I | Session II | Session III | Session IV | Session V | Session VI | Comment phase | Session VII |
| Activities | • Discussion of goals, scope, and working group members' perspectives on selection criteria | • Agreement on selection criteria<br>• Agreement of key concepts<br>• First discussion of PRO(s) | • PRO operationalization<br>• PROM introduction | • Final selection of PROs<br>• Agreement on PROM selection criteria | • Update on PROM quality evaluation<br>• Review of umbrella & patient organizations feedback | • Final selection of PROMs<br>• Discussion on recommendations for implementation and use | • Webinar for stakeholders<br>• Submission of preliminary results to umbrella organizations | • Discussion of stakeholder feedback<br>• Amendment of PROM selection and recommendations for use<br>• Approval by board of umbrella organisations on January 13, 2022 |

**Supplemental Table 1. PROMs signaling questions**

| Domain | Quality criteria | Green rating | Amber rating | Red rating |
|---|---|---|---|---|
| Feasibility | Number of items | < 20 items | 20-25 item | >25 items |
| | Patient friendliness* | CEFR Language Level <B2 AND<br>< 20 items AND<br>< 5 answer options in rating scale or numeric 0-10 rating scale AND<br>>= 67% 'green' ratings on remaining items | Remaining cases | CEFR Language Level =C1 OR<br>> 25 items OR<br>> 5 options in rating scale OR<br><50 % 'green' ratings on remaining items |
| | Costs | No | Yes | Not applicable |
| Content validity | Criterion 1: 85% of the items are relevant to measure the construct<br>Criterion 2: The set of items provides a comprehensive assessment of the subdomains of the construct | Positive expert judgement for both criteria based on agreement among two raters | Remaining cases | Negative expert judgement for one or both criteria based on agreement among two raters |
| Construct validity** | Hypothesis about correlation with other outcomes OR core differences between groups known to differ with respect to the construct to be measured | >= 75% of hypotheses (n >5) of correlation with other outcome measurements or of differences scores between known groups is confirmed. | Remaining cases | 50% of hypotheses (n >5) of correlation with other outcome measurements or of difference scores between known groups is confirmed. |
| Reliability** | Test retest or inter-item relations based reliability coefficient | Lowest reliability found >0.7, 2 or more studies | Remaining cases | Lowest reliability found <0.7, 2 or more studies |
| Responsiveness** | Change scores consistent with hypotheses with respect to change in clinical status | Change scores consistent with hypotheses, 2 or more studies | Remaining cases | Change scores inconsistent with hypotheses, 2 or more studies |

*Assessed using the Pharos rapid test for patient questionnaires in healthcare.; Criteria adapted from the criteria for good measurement properties listed in the COSMIN manual for systematic reviews of PROMs

**Supplemental Table 2. PRO identification**

| Domain | Disease impact level | Initial categorization of PROs | Refined categories | Voting results* working group members (n=14) | Voting results *patient organizations (n=8) |
|---|---|---|---|---|---|
| Pain | Symptoms | Symptoms | Fatigue | 9 (64%) | 7 (88%) |
| Fatigue | | | | | |
| Sleep | | | Sleep | 4 (29%) | 3 (38%) |
| Sight | | | | | |
| Vitality | | | | | |
| Appetite/weight loss | | | Pain | 9 (64%) | 7 (88%) |
| Symptom burden | | | | | |
| Hearing problems | | | | | |
| Daily activities | Functioning | Physical functioning | Physical functioning | 14 (100%) | 8 (100%) |
| Problem solving | | | | | |
| Self-efficacy | | | | | |
| Mobility | | | | | |
| Physical functioning | | | | | |
| Work | | | | | |
| daily life independence | | | | | |
| General physical function | | | | | |
| Healthy lifestyle | | | | | |
| Work | | | | | |
| Daily activities | | | | | |
| Mobility | | | | | |
| Mobility restrictions | | | | | |
| Cognition | | Mental functioning | Anxiety | 2 (21%) | 2 (25%) |
| Depression | | | | | |
| Anxiety | | | | | |
| Anxiety & Depression | | | Cognition | 2 (14%) | 3 (38%) |
| General mental health | | | | | |
| Depression | | | | | |
| Emotional health | | | Mental | 10 (71%) | 7 (88%) |

| | | | | | |
|---|---|---|---|---|---|
| Concentration/memory | | | | | |
| Cognitive functioning | | | | | |
| Mental Health | | | Depression | 3 (21%) | 1 (13%) |
| Anxiety | | | | | |
| Mental health | | | | | |
| Social roles | | Social functioning | Social functioning/participation | 13 (93%) | 6 (75%) |
| Social activities | | | | | |
| Interpersonal functioning | | | | | |
| Participation | | | | | |
| General Social Health | | | | | |
| Social contacts | | | | | |
| Sexuality | Remaining items | Sexuality | Sexuality | 4 (29%) | 1 (13%) |
| sexual functioning | | | | | |
| Intimate relations | | | | | |
| Health experience | | Overarching | Perceived Overall Health | 10 (71%) | 5 (63%) |
| General Physical Health | | | | | |
| Overall Health Experience | | | | | |
| General Health | | | | | |
| Overall sense of health | | | | | |
| Quality of life | | | Quality of life | 9 (64%) | 7 (88%) |
| Meaning | | | | | |
| What is important in life | | | | | |
| Valued activities | | | | | |
| Quality of life | | | | | |

***= N (%) of raters including the PRO in top 7 of most important outcomes**

**Supplemental Table 3. Assessment of PROMs**

| PRO(s) | PROM(s) | Content validity | Feasibility | | | Measurement properties | | | PROMIS Metric |
|---|---|---|---|---|---|---|---|---|---|
| | | | Burden | Items | Costs | Reliability | Validity | Responsiveness | |
| **Quality of life** | Topics NRS Quality of life | + | ? | + | + | ? | ? | ? | n.a. |
| | V1.2 PROMIS Global02 | + | ? | + | + | ? | ? | ? | n.a. |
| **Perceived health** | PROMIS Global 01 | + | ? | + | + | ? | ? | ? | n.a. |
| | SF-36 question 1 | + | ? | ? | + | ? | ? | ? | n.a. |
| | TOPICS-SF NRS Overall health | ? | + | + | + | ? | ? | ? | n.a. |
| **Participation in social roles** | WHO Disability Assessment Scale (WHODAS) Participation | - | ? | + | + | + | + | ? | ? |
| | SF-36/RAND-36 Emotional role functioning | + | - | + | + | + | + | + | ? |
| | SF-36/RAND-36 Physical role functioning | + | ? | + | + | + | + | + | ? |
| | V2.0 PROMIS Ability to Participate in Social Roles and Activities SF4a | + | ? | + | + | + | ? | ? | + |
| | V2.0 PROMIS Ability to Participate in Social Roles and Activities SF6a | + | ? | + | + | + | ? | ? | + |
| | V2.0 PROMIS Ability to Participate in Social Roles and Activities SF8a | + | ? | + | + | + | ? | + | + |
| | V2.0 PROMIS Ability to Participate in Social Roles and Activities CAT | + | ? | ? | + | + | ? | + | + |
| | TOPICS-SF 4 point rating scale social activities | - | ? | + | + | ? | ? | ? | ? |
| | Utrecht Scale for Evaluation of Rehabilitation-Participation (USER-Participation restrictions | + | ? | + | + | ? | ? | ? | ? |
| | Positive Health Questionnaire Participation | - | + | ? | + | ? | ? | ? | ? |

| | | | | | | | | | |
|---|---|---|---|---|---|---|---|---|---|
| | Functional Assessment of Chronic Illness Therapy - Participation (FACIT-P) | - | ? | + | + | ? | ? | ? | ? |
| **Physical functioning** | SF-36/RAND-36 Physical Functioning | + | + | + | + | + | + | + | + |
| | TOPICS-SF Tasks and activities of daily life | ? | ? | + | + | ? | ? | ? | ? |
| | Positive Health Questionnaire Everyday activities | - | + | + | + | ? | ? | ? | ? |
| | V2.0 PROMIS Physical Function SF4a | - | ? | + | + | + | + | ? | + |
| | V2.0 PROMIS Physical Function SF6b | - | ? | + | + | + | + | ? | + |
| | V2.0 PROMIS Physical Function SF8b | - | ? | + | + | + | + | ? | + |
| | V2.0 PROMIS Physical Function SF10a | + | ? | + | + | + | + | + | + |
| | V2.0 PROMIS Physical Function SF10b | + | ? | + | + | + | + | + | + |
| | V2.0 PROMIS Physical Function SF20a | + | ? | + | ? | + | + | + | + |
| | V1.2 PROMIS Physical Function CAT | + | ? | ? | + | + | + | + | + |
| **Anxiety** | GAD-7 | + | ? | + | + | + | ? | ? | + |
| | 4DKL Anxiety | + | ? | ? | + | ? | ? | ? | ? |
| | HADS Anxiety | + | ? | ? | + | + | + | + | + |
| | MASQ | - | ? | + | + | ? | ? | ? | + |
| | V1.0 PROMIS Anxiety short form 4a | + | ? | + | + | ? | + | + | + |
| | V1.0 PROMIS Anxiety short form 6a | + | ? | + | + | ? | + | + | + |
| | V1.0 PROMIS Anxiety short form 8a | + | ? | + | + | + | + | + | + |
| | V1.0 PROMIS Anxiety short form | + | ? | + | + | + | + | + | + |

| | | | | | | | | | |
|---|---|---|---|---|---|---|---|---|---|
| | 7a | | | | | | | | |
| | V1.0 PROMIS Anxiety CAT | + | ? | + | + | + | + | + | + |
| **Depression** | PHQ-9 | ? | ? | + | + | + | ? | ? | + |
| | PHQ-2 | ? | ? | + | + | + | + | ? | + |
| | V1.0 PROMIS Depression short form 4a | ? | ? | + | + | + | + | + | + |
| | V1.0 PROMIS Depression short form 6a | ? | ? | + | + | + | + | + | + |
| | V1.0 PROMIS Depression short form 8a | ? | ? | + | + | + | + | + | + |
| | V1.0 PROMIS Depression short form 8b | ? | ? | + | + | + | + | + | + |
| | V1.0 PROMIS Depression CAT | ? | ? | ? | + | + | + | + | + |
| | HADS Depression | - | ? | ? | + | + | + | + | + |
| | WHO-5 Wellbeing Index | - | - | + | + | ? | ? | ? | ? |
| | Beck Depression Inventory-II (BDI-II) | ? | ? | ? | ? | + | ? | ? | + |
| | 4DKL Depression | + | ? | ? | ? | ? | ? | ? | ? |
| | Positive Health Questionnaire – Mental wellbeing | - | + | + | + | ? | ? | ? | ? |
| | CES-D | + | ? | ? | ? | ? | + | ? | + |
| **Fatigue** | Numeric Rating Scale (NRS-11) fatigue | + | + | + | + | ? | ? | ? | + |
| | Functional Assessment of Chronic Illness Therapy - Fatigue (FACIT-F) | - | ? | ? | + | + | + | ? | + |
| | Fatigue Severity Scale (FSS) | - | - | ? | + | ? | ? | ? | ? |
| | Modified Fatigue Impact Scale (MFIS) | - | ? | ? | ? | ? | ? | ? | ? |
| | V1.0 PROMIS Fatigue short form 4a | + | ? | + | + | + | + | + | + |
| | V1.0 PROMIS Fatigue short form 6a | ? | ? | + | + | + | + | + | + |
| | V1.0 PROMIS Fatigue short form | ? | ? | + | + | + | + | + | + |

| | | | | | | | | | |
|---|---|---|---|---|---|---|---|---|---|
| | 8a | | | | | | | | |
| | V1.0 PROMIS Fatigue CAT | ? | ? | ? | + | + | + | + | + |
| **Pain** | NRS Pain Intensity | + | + | + | + | ? | + | ? | n.a. |
| | PROMIS® Numeric Rating Scale v1.0 – Pain Intensity 1a | + | + | + | + | ? | ? | ? | n.a. |
| | SF-36/RAND-36 Bodily Pain | ? | - | + | + | + | + | + | n.a. |
| | TOPICS-SF 4 point Rating scale pain | ? | ? | + | + | ? | ? | ? | n.a. |

'+' = 'Green rating'; see Supplemental Table 1. PROMs signaling questions , "-" = 'Red rating'; see Supplemental Table 1. PROMs signaling questions – '?'= 'Amber rating'; see Supplemental Table 1. PROMs signaling questions
